# SpheroMold: modernizing the hanging drop method for spheroid culture

**DOI:** 10.3389/fddev.2024.1397153

**Published:** 2024-06-05

**Authors:** Ana Paula Pereira Guimaraes, Italo Rodrigo Calori, Hong Bi, Antonio Claudio Tedesco

**Affiliations:** ^1^ Department of Chemistry, Center of Nanotechnology and Tissue Engineering- Photobiology and Photomedicine Research Group, Faculty of Philosophy, Sciences and Letters of Ribeirao Preto, University of Sao Paulo, Sao Paulo, Brazil; ^2^ Pharmaceutical Engineering and 3D Printing (PharmE3D) Labs, Department of Pharmaceutics and Drug Delivery, School of Pharmacy, The University of Mississippi, Oxford, MS, United States; ^3^ School of Materials Science and Engineering, Anhui University, Hefei, China; ^4^ Anhui Key Laboratory of Modern Biomanufacturing, School of Chemistry and Chemical Engineering, Anhui University, Hefei, China

**Keywords:** PDMS, 3D printing, spheroid, 3D cell culture, hanging drop, stereolithography

## Abstract

The hanging drop method is a cost-effective approach for 3D spheroid culture. However, obtaining numerous spheroids in a limited area becomes challenging due to the risk of droplet coalescence, primarly during Petri dish handling. In this study, we describe a general method to fabricate a 3D printing-based support called SpheroMold that facilitates Petri dish handling and enhances spheroid production per unit area. As a proof-of-concept, we designed a digital negative mold which comprised 37 pegs within a 13.52 cm^2^ area, and then printed it using stereolithography; the density of pegs can be adjusted according to user requirements. The SpheroMold was created by pouring the base and curing agent (10:1) (Sylgard^®^ 184 silicone) into the mold, curing it at 80°C, and then attaching it to the lid of a Petri dish. Our SpheroMold effectively prevented droplet coalescence during Petri dish inversion, enabling the production of numerous 3D spheroids while simplifying manipulation. Unlike conventional techniques, our design also facilitated a larger volume of culture medium per drop compared to a standard Petri dish, potentially decreasing the necessity for frequent medium exchange to sustain cellular health and reducing labor intensity.

## 1 Introduction

Three-dimensional (3D) multicellular spheroids play an important role in advancing our understanding of cellular behavior, disease mechanisms, and drug responses, in a context with greater physiological relevance than conventional two-dimensional cell cultures (Gayan et al., 2017; Pingitore et al., 2019; Ding et al., 2022; Oh et al., 2022). Researchers have recognized the potential of 3D spheroids to bridge the gap between simple cell monolayers and the complex 3D environments of living organisms. From this perspective, there is a growing interest in establishing consistent and user-friendly methodologies for 3D spheroids (Park et al., 2017; Kahn-Krell et al., 2022; Pulugu et al., 2022). This is even more crucial, given the urgent need for alternative animal models based on the 3R principles of preclinical research (Replacement, Reduction, and Refinement) (Franco et al., 2018; Fröhlich and Loizou, 2023).

The hanging drop method is a cost-effective approach that requires minimal tools to produce 3D spheroids (Shao et al., 2020). The method involves depositing droplets of culture medium containing cells on the bottom of a culture Petri dish lid, flipping it over onto the Petri dish, and incubating under normal cell culture conditions. Due to gravity, cells aggregate around the lowest point of the droplet, forming spheroids after a few days of culture. This simple procedure yields spheroids of relatively uniform size and shape. Compared to certain protocols such as agitation and magnetic techniques, the hanging drop method minimizes mechanical stress on cells by eliminating the need for external forces, enabling a more natural self-assembly of cells into 3D structures. Moreover, the versatility of the hanging drop method, which is applicable to various cell types, makes it a viable option for a broad range of research goals (Kelm et al., 2003; Monico et al., 2022).

Despite its advantages, the hanging drop method encounters challenges when cultivating numerous spheroids in a limited plate area. The delicate process of inverting the plate lid, crucial for medium replacement, spheroid imaging, and drug testing, carries a significant risk of droplet fusion due to the proximity of adjacent drops. In addition, plate manipulation can also cause droplet dripping and loss of shape. This concern becomes more pronounced when Petri dishes are reused, leading to increased droplet dispersion. Additionally, the small volume of culture medium per droplet requires frequent replenishment to prevent nutrient deficiency, thereby increasing the labor-intensive nature of droplet manipulation.

Using a matrix to position and confine droplets on the plate could enhance spheroid production in a limited area via the hanging drop method. Additionally, this matrix facilitates plate manipulation processes, reducing the need for careful handling to prevent droplet coalescence. Furthermore, the matrix can be designed to confine a larger volume of medium per droplet, potentially reducing the frequency of culture medium renewal.

**FIGURE 1:**
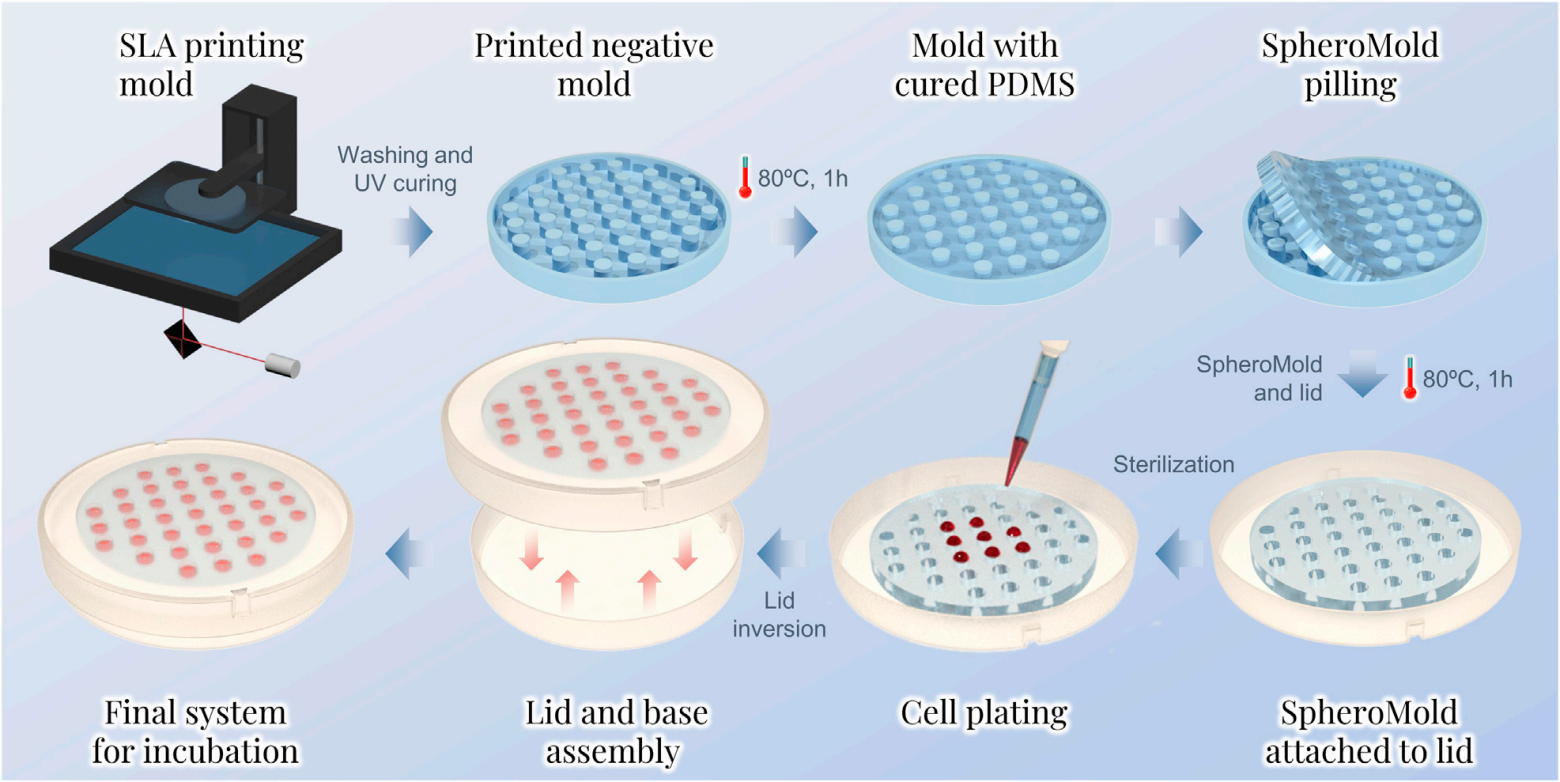
Sequential steps for the spheroid preparation by the hanging drop method using the SpheroMold.

Herein, we introduce a 3D printing-based support, called SpheroMold, designed to accommodate numerous drops in a limited area, as shown in Figure 1. The 3D printed SpheroMold was made of polydimethylsiloxane (PDMS) to avoid any cell toxicity. The precisely positioned holes and tight spacing between them on the SpheroMold increased the drop count per plate. As a proof-of-concept, within 13.52 cm^2^, 37 drops were incorporated. Well-defined openings in the SpheroMold were demonstrated to enhance droplet generation efficiency, enabling precise medium addition in close and specific spots of the Petri dish lid. Additionally, the physical barrier of the holes aids in plate manipulation and inversion, preventing runoff and the merging of adjacent drops. Furthermore, the thickness of the openings allows for a greater medium volume, thereby reducing the need for frequent medium renewal to maintain cell viability.

## 2 Materials and methods

### 2.1 Materials section

The base and curing agent Sylgard 184 kit were purchased from Dow Corning (Dow Corning, Midland, Michigan, USA). The human glioblastoma U-251 MG cell line and isopropyl alcohol were purchased from Sigma Aldrich (Sigma-Aldrich, San Luis, MO, United States). Modified Dulbecco’s Eagle Medium (DMEM), amphotericin B, penicillin, streptomycin, fetal bovine serum (FBS), and the live/dead assay kit were purchased from Life Technologies (ThermoFisher Scientific Inc., Waltham, MA, USA). The Milli-Q^®^ system was used to prepare ultrapure water, which was utilized in all experiments.

### 2.2 SpheroMold preparation

For the negative mold, a. STL file was designed via 3DS Max 2023 software. The model was 3D printed using photopolymer resin on an ELEGOO Mars 2 Pro printer. After printing, the negative mold was cleaned with isopropyl alcohol to eliminate uncured residues. The cleaned mold was then exposed to UV light until fully cured. To facilitate the PDMS curing, a spray varnish was applied to the mold. After 24 h of varnish application, Sylgard 184 (10:1 pre-polymer-to-curing-agent ratio) was poured into the mold cavities. The mixture was cured at 80°C for 1 h. Next, the SpheroMold was carefully removed from the mold and attached to a Petri dish lid. This attachment involved applying a thin layer of uncured Sylgard mixture between both objects followed by curing (80°C, 1 h). Before use in cell culture, the matrix-containing lid was sterilized with formaldehyde gas.

### 2.3 Inversion plate assay

Droplets of 10, 15, and 20 µL of culture medium were added to the lid of a Petri dish; a SpheroMold was positioned below the lid as a reference to guide the droplet placement. The dish was inverted ten times to simulate the standard lid inversions performed in hanging drop spheroid assays. Photographic images were captured after every two inversions, followed by the analysis of droplet shape, spreading, fusion, and counting using ImageJ.

### 2.4 Contact angle

The contact angle of the suspended droplets was measured using an OCA 20 instrument (Dataphysics Instruments Inc., Filderstadt, DE) assisted by SCA 20 software (Dataphysics Instruments Inc., Filderstadt, DE). Initially, droplets of the culture medium of various volumes were introduced into the holes of the lid containing the SpheroMold. The lid was then inverted. The mean contact angle was determined 120 s after inversion. All samples were maintained under standard room conditions during the experiment. The final value was accepted as the average of three separate measurements.

### 2.5 Cell culture

The Glioblastoma U251 cell line was cultured in DMEM supplemented with 10% fetal bovine serum (FBS), 100 U/mL penicillin, 100 mg/mL streptomycin, 250 ng/mL amphotericin B, and 0.1 mmol/L MEM non-essential amino acids. The cell line was cultivated in a controlled incubator at 37°C with 5% CO_2_ and controlled humidity.

### 2.6 Cell spheroid formation

U251 cell spheroids were formed based on the hanging drop method. Initially, 35 μL droplets of cell-containing culture medium with either 500 or 2000 cells were placed into each hole of the SpheroMold attached to the lid. The lid was then inverted onto the base of a dish containing 5 mL of PBS. The plate was subsequently incubated at 37°C with 5% CO_2_ and controlled humidity for up to 5 days. Controls were established using the matrix-free and PDMS-coated bases of the Petri dish lid.

### 2.7 Cell viability assay

Cell viability was evaluated using a live/dead kit to distinguish between live and dead cells within the spheroids. Spheroids cultured for 5 days were placed on a coverslip and washed twice with PBS. Then, the spheroids were exposed to a mixture of 2 × 10^−6^ mol/L ethidium homodimer-1 and 1 × 10^−6^ mol/L calcein AM for 15 min at 37°C. After two washes with PBS, confocal images were acquired using a Leica SP8 Laser confocal microscope.

## 3 Results and discussion

The SpheroMold was designed with symmetrically distributed cylindrical holes along a circular PDMS base of 13.52 cm^2^, as illustrated in Figure 1. The distance between holes in the SpheroMold was selected to create 37 drops equally distributed along the base. Decreasing the hole distance could be employed to enhance the number of holes per matrix; however, matrices configured under this condition amplify the demolding challenge from the negative mold, particularly with thicker bases.

**FIGURE 2:**
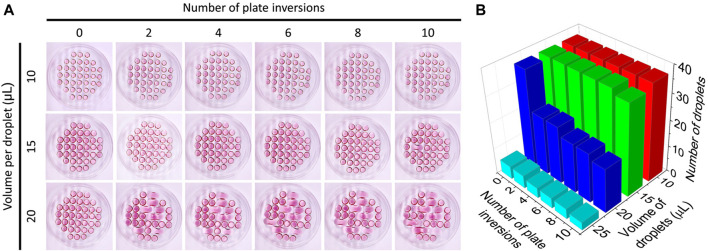
**(A)** Culture medium droplet arrangement of culture medium at different volumes on the lid of a Petri dish as a function of the number of times the plate was inverted. **(B)** Number of intact droplets as a function of volume and number of times the lid was inverted.

To assess the limitations of conventional Petri dishes in maintaining the droplet density proposed in the SpheroMold, cell-free drops of various volumes were added on a matrix-free Petri dish lid. The droplets on the lid followed the same arrangement as that of the SpheroMold (Figure 2A). Using 10 μL, none of the drops exhibited fusion after plating. Moreover, droplet integrity was maintained even after up to ten lid inversions were performed. A similar result was obtained with a 15 μL volume. However, the inversion steps appeared to be more critical, as in some instances, drop fusion occurred after the 10th inversion. More critically, when utilizing a 20 μL volume, fusion of certain drops occurred typically within the initial two inversions. This occurrence became increasingly frequent with successive inversions (Figure 2B). It's worth highlighting that a volume surpassing 20 μL hindered the accurate formation of drops at their designated positions on the 3D printed SpheroMold. While it is evident that these values can vary due to differences in plate manipulation techniques, this data underscores that, to a certain extent, handlers may encounter challenges when dealing with a medium volume higher than 15 μL.

**FIGURE 3:**
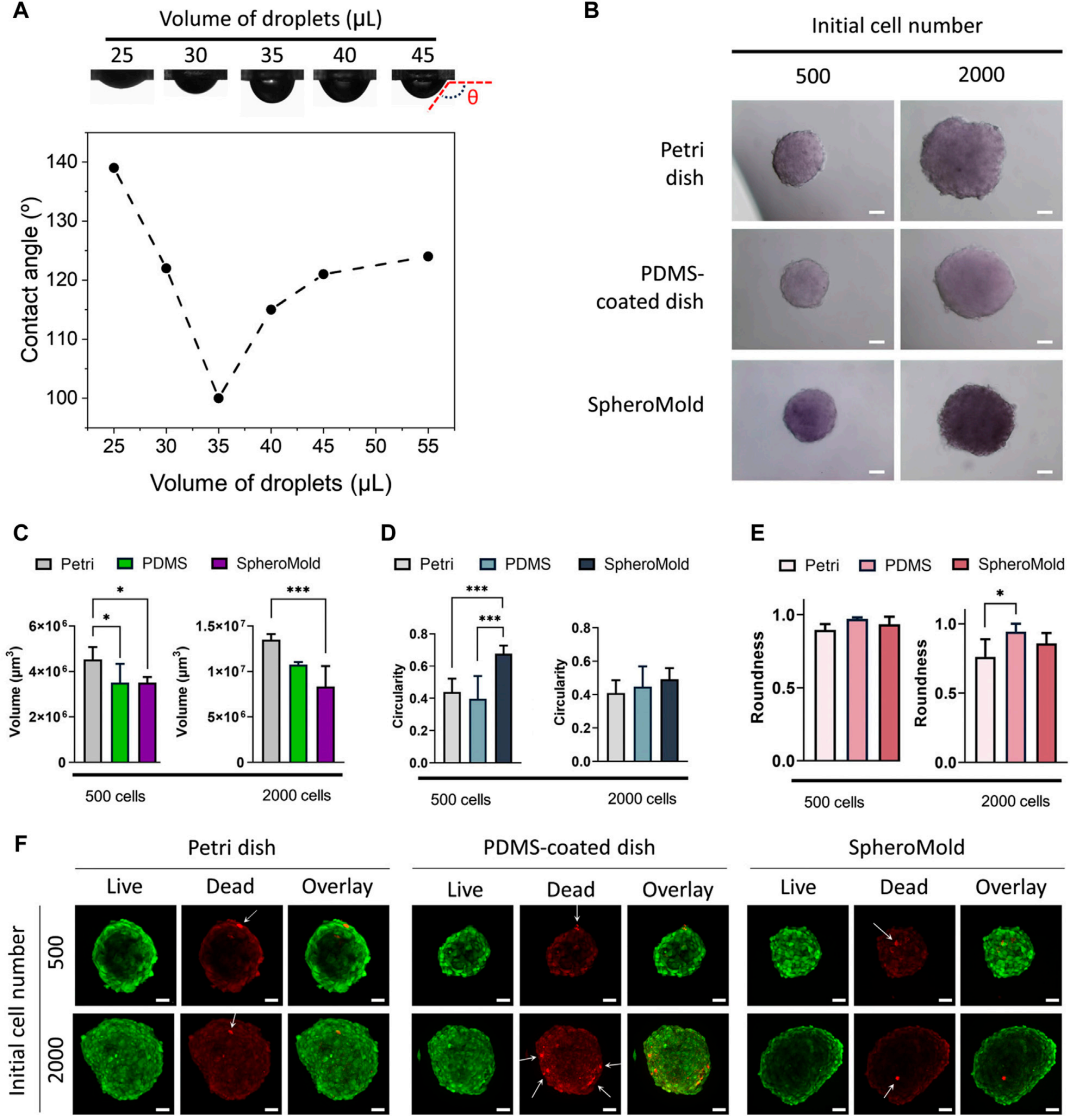
**(A)** Photograph images and contact angle of culture medium droplets on SpheroMold as a function of droplet volume. **(B)** Optical images, **(C)** volume **(D)** circularity, **(E)** roundness, and **(F)** live/dead confocal images of U251 spheroids (500 and 2000 cell per spheroid) on day 5 generated in Petri dishes, PDMS-coated dish, and SpheroMold. Optical and confocal images were acquired using a ×20 objective.

In comparison to the results obtained using conventional lids of Petri dishes, our designed SpheroMold facilitated the accommodation of up to 35 μL of medium, as evidenced by the minimum contact angle value in Figure 3A. With a volume of 35 μL, the SpheroMold successfully prevented drops from dripping or fusing during plate inversions. In contrast to the conventional Petri dishes, which displayed some droplet dispersion upon reuse (not shown), the reutilization of the matrix was reproducible. This implies that the SpheroMold is well-suited for reuse following sterilization methods, such as formaldehyde gas application. Moreover, the capability to manage elevated volumes without compromising droplet integrity, coupled with improved maneuverability during plate inversions, represents notable progress in the optimization of spheroid culture conditions using hanging drop methods.

Volumes exceeding 35 μL were possible to use; however, some dispersion of drops beyond the boundary of the hole, partially spreading on the surface of PDMS, was observed. This finding was also demonstrated by the increased contact angles determined using these volumes. Nevertheless, there were no collisions with adjacent drops. In this way, drops were maintained using up to 50 μL of medium. Notably, the ability to accommodate larger volumes of the medium per drop can be adjusted by changing the depth of the holes within the matrix. These findings highlight the superiority of the matrix in terms of medium capacity and handling compared to conventional lids of Petri dishes.

Motivated by the outstanding results achieved using the 3D printed SpheroMold, spheroid formation was evaluated to assess the effectiveness of the SpheroMold. U251 cells were used for this purpose. This particular cell line is recognized for its ability to self-form spheroids on nonadherent round-bottom plates (Calori et al., 2022; Alves et al., 2023). Spheroid cultures were established using 500 and 2000 cells in a 3D printed SpheroMold. The PDMS-free and PDMS-coated lids were used as controls. Optical images revealed similarities among the spheroids generated using all the systems (Figure 3B). After 5 days of cultivation, all spheroids exhibited tight compaction and notable sphericity. The use of PDMS, particularly with the SpheroMold, reduced the volume of the spheroids (Figure 3C). This suggests that our SpheroMold may produce more compact spheroids, compatible with solid glioblastoma tumors. Additionally, the SpheroMold improved circularity (Figure 3D), especially for spheroids of 500 cells. Roundness values were consistently high across all systems, showing no significant difference (Figure 3E). The capacity of the SpheroMold to form 3D spheroids could have substantial implications for applications that demand a substantial quantity of well-defined and reproducible spheroids while utilizing a limited number of plates.

The live/dead data revealed that spheroids fabricated using the SpheroMold showed similar or higher cell viability than those produced using control methods (Figure 3F). Only a limited number of dead cells were observed in all systems used, as indicated by the white arrows in Figure 3C; it is worth noting that the red background signal observed is due to the residual emission from the live cell dye and does not represent any dead cells.

Taken together, the findings emphasize the effectiveness of the SpheroMold in producing tight, compact, and viable cell spheroids, combined with the additional advantages of easy handling and a higher number of spheroids. This facilitates manipulative procedures, leading to reduced labor time and improved efficiency. Although the process of printing a negative mold requires substantial labor from the researchers, the ability of the plate to be reused several times leads to savings in terms of both time and finances, a benefit that cannot be attained when using traditional Petri dishes.

## 4 Conclusion

In this study, we introduced a novel setup for a 3D printed SpheroMold that effectively augmented spheroid production and facilitated manipulation procedures using the hanging drop method. The setup exhibited superior performance compared to conventional plates, effectively maintaining drop integrity and preventing undesirable fusion during plate manipulation. This setup facilitated an increased spheroid yield within a limited plate area. The resultant spheroids demonstrated compaction and sphericity comparable to those cultivated using traditional plates, while maintaining viability. A larger volume of medium per droplet supported by the matrix, compared to the traditional Petri dish, could ensure a reduced manipulation frequency, thereby decreasing the labor-intensive nature of spheroid production. These findings provide substantial evidence for the effectiveness of our approach, concurrently simplifying handling processes and reducing labor duration. This pioneering 3D printed SpheroMold represents a significant advancement in addressing the challenges related to spheroid manipulation through the hanging drop method, as it provides a larger number of spheroids per Petri dish lid area, improved control over plate inversion, and a lower frequency of medium replacement.

## Data Availability

The original contributions presented in the study are included in the article, further inquiries can be directed to the corresponding author.
